# Acute Myeloid Leukemias with Alterations of Lysine Methyltransferase 2A (KMT2A): Recent Therapeutic Developments

**DOI:** 10.3390/cancers18091341

**Published:** 2026-04-23

**Authors:** Ugo Testa, Elvira Pelosi, Germana Castelli

**Affiliations:** Department on Oncology, Istituto Superiore di Sanità, Viale Regina Elena 299, 80122 Rome, Italy; elvira.pelosi56@gmail.com (E.P.); germana.castelli@iss.it (G.C.)

**Keywords:** acute myeloid leukemia, *KMT2A* rearrangements, Menin inhibitors, targeted therapy, intensive chemotherapy, *HOX* genes

## Abstract

*Background*: Chromosomal rearrangements involving lysine methyltransferase 2A (KMT2A) define a genetically distinct subset of acute myeloid leukemia (AML) in 10% of cases in adult patients; the frequency of *KMT2A-r* is higher in pediatric AML. Translocations involving the *KMT2A* locus at chromosome 11q23 result in the formation of a chimeric oncogene partner, where the N-terminal part of *KMT2A* is fused to a variety of translocation partners. The leukemogenic activity of *KMT2A*-fusion partners is related to their capacity to hyperactivate the expression of *HOX-A* and *MEIS1* target genes, which stimulate the proliferation of hematopoietic stem cells. The oncogenic activity of *KMT2A* fusion proteins requires the binding with Menin, and this interaction can be targeted pharmacologically by small molecules acting as potent and selective Menin inhibitors. *Methods*: A search of the literature showed a marked development of experimental studies exploring the molecular pathogenesis of AML with *KMT2A-r* and of clinical studies evaluating new induction intensive treatments and the development of a targeted therapy based on Menin inhibitors. *Results and Conclusions*: In the present review article, we summarize our current understanding of the biology of *KMT2A-r* in AML development and the recent consistent progress made in the treatment of *KMT2A-r* AML through new chemotherapy regimens and targeted therapy using Menin inhibitors. However, the prognosis of older *KMT2A-r* AML patients remains poor and could be improved by drug combination studies including Menin inhibitors. Many encouraging observations derived from ongoing clinical trials with Menin inhibitors need to be confirmed through randomized clinical trials.

## Simple Summary

KMT2A-rearranged (*KMT2A-r*) acute myeloid leukemia (AML), previously known as mixed lineage leukemia (MLL), is a high-risk, aggressive AML subtype characterized by chromosomal translocations involving the *KMT2A* gene on chromosome 11q23 and by high expression of *HOX-A* and *MEIS1* genes. These rearrangements account for 5–10% of de novo adult AML cases and are associated with poor prognosis. The aim of this review is to provide a detailed analysis of recent developments in understanding the molecular mechanisms underlying leukemic development driven by *KMT2A-r* and in the treatment of these AML through new intensive regimens and targeted therapy using a new category of antileukemic drugs, Menin inhibitors.

## 1. Introduction

Acute myeloid leukemia (AML) is a heterogeneous hematologic malignancy characterized by clonal expansion, uncontrolled proliferation, and differentiation arrest of myeloid progenitor cells. AML is highly heterogeneous at the molecular level and is characterized by multiple somatic genetic events, some of them acting as driver genetic events.

The lysine methyltransferase 2A gene (*KMT2A*), a histone 3 lysine 4 methyltransferase, previously known as mixed lineage leukemia (MLL), plays a key role in the control of normal hematopoiesis, and its alterations are frequently observed in several hematologic malignancies.

In the World Health Organization (WHO) classification, AML with *KMT2A-r* is defined as a distinct clinical entity in the presence of any *KMT2A* rearrangement [1]. The European Leukemia Net (ELN) guidelines generally classify t(9;11)/*KMT2A::MLLT3* as intermediate-risk and other *KMT2a-r* cases as adverse-risk [2]. At variance with WHO 2022, the International Consensus Classification (ICC) 2022 specifically separates t(9;11)/*KMT2a::MLLT3* as a distinct entity from other *KMT2A* rearrangements [3]. Rearrangement of the *KMT2A* gene in AML results in the generation of fusion proteins that cause epigenetic dysregulation of hematopoietic cells and upregulation of *HOXA*, *HOXB,* and *MEIS1* genes, leading to leukemia. AML with *KMT2A-PTD* is increasingly recognized as a distinct molecular entity in AML, due to its unique genomic structure (frequent *DNMT3A*, *FLT3-ITD*, *IDH1*, and *IDH2* mutations) and clinical behavior, associated with distinct gene signatures (high *HOX* gene expression) and adverse prognosis [4].

Three types of *KMT2A* gene alterations may be observed in AML: (i) rearrangements of the *KMT2A* gene involving events of translocations between the *KMT2A* gene and a partner gene with formation of a fusion gene and a corresponding fusion protein composed by the N-terminus of the *KMT2A* gene fused in frame to one of many different partners; (ii) *KMT2A-PTD*, involving the duplication of *KMT2A* gene segment comprised between exons 2 to 9; (iii) point mutations of the *KMT2A* gene [5]. A recent study carried out at the Cleveland Clinic, Ohio, USA, explored *KMT2A* gene alterations in 730 adult AML patients, showing 88% of patients with *KMT2A*-WT and 12% with *KMT2A* alterations: 5.7% with *KMT2A-r*, 3.2% with *KMT2A* point mutation, and 2.3% *KMT2-PTD* [5]. Other studies confirmed that most of the alterations of the *KMT2A* gene are large rearrangements, with fusions being the most commonly observed alterations, with no amplifications or deletions observed [6].

The aim of the present review is to provide an overview of the recent progress made in the understanding of the molecular pathogenesis, in the biological and clinical characterization, and in the response to therapy of AML with *KMT2A* abnormalities, including recent developments in newer targeted therapies.

## 2. KMT2A Gene and Protein Structure

The *KMT2A* gene family consists of seven conserved histone 3 lysine 4 (H3K4) methyltransferases that act as critical epigenetic regulators. The histone methyltransferase 2 family comprises a highly conserved group of histone methyltransferase enzymes that are involved in mono-, di-, and tri-methylation at histone three lysine 4 through the enzymatic activity of their conserved SET domain [7]. Six mammalian KMT2 proteins of three subgroups, KMT2A/B (MLL 1–2), KMT2 C/D (MLL 3–4), and KMT2 F/G (SETD1 A/B), have shared and distinct protein domains, catalytic substrates, genomic locations, and associated complex subunits [7]. The C-terminal SET catalytic domain of KMT2A confers mono, di-, and tri-methylation on histone H3K4. These enzymes function within large multi-protein complexes known as COMPASS (Complex of Proteins Associated with SET1). The function of KMT2 enzymes is fundamental for the activation of gene transcription, for development, and for hematopoiesis. *KMT2* genes are frequently mutated in cancers.

The *KMT2A* gene comprises 38 exons, including the 5′ and 3′-untranslated regions (UTR), distributed across a 90,375 bp region at 11q23.3. The major breakpoint cluster region (BRC1) is located at the level of a DNA sequence comprised between *KMT2A* exons 9 and 12 (>90% of *KMT2A* rearrangements), while a minor breakpoint cluster region (BCR2) is mostly located between exons 21 and 25 [8].

**Figure 1:**
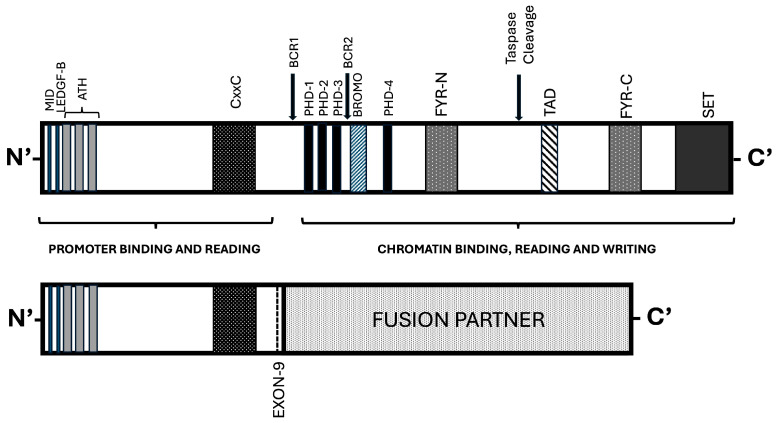
Top panel: Structure of the WT KMT2A protein. Bottom panel: KMT2A fusion protein formed through gene rearrangement events. The main structural elements of the KMT2A protein are reported, and their function is analyzed in the text.

KMT2A is a 500 kDa protein and acts as a key epigenetic regulator that exerts its biological activity as a “writer” of histone markers, to control gene expression during hematopoiesis, embryonic development, and neurodevelopment. The KMT2A protein is organized at the structural level with a modular design, with molecular regions involved in mediating DNA binding, protein-protein interactions, and enzymatic activity (Figure 1). The full-length KMT2A protein is cleaved by the endopeptidase Taspase 1 into two different subunits: KMT2A N-terminal (KMT2AN) and KMT2A-C-terminal (KMT2AC); these two subunits are non-covalently linked at the level of FYRNR and FYRC domains and exert their function together as a heterodimer [9]. The N-terminal subunit of KMT2A protein contains several functional domains mainly represented by: binding DNA sequences for Menin (MID) and LEDGF (Lens Epithelium Derived Growth Factor), required for guiding the complex between KMT2A and Menin, using LEDGF proteins to target genes; three AT-hooks required for DNA binding; and a CxxC domain, known also as MBD, required to promote binding of unmethylated CpG islands located in gene promoters [8,9]. The C-terminal domain contains two functional domains: the catalytic SET domain, which possesses methyltransferase activity, and the TAD domain, which interacts with the histone acetyltransferase CBP/p300, MD2, and MOF to enable appropriate histone acetylation [8,9] (Figure 1). The N-terminal domain also contains two sets of regulatory domains: Plant Homeodomain (PHD) and Bromodomain (BRD). PHDs are zinc finger structures that function as chromatin readers, guiding the KMT2A methyltransferase enzyme to specific active genomic regions. BRD functions as an epigenetic reader that recognizes acetylated lysine residues on histones, enhancing the interaction of the adjacent PHD domain with the H3K4me3 mark [8,9].

The main biological function of KMT2A consists of acting as an epigenetic writer that catalyzes the mono-, di-, and tri-methylation of histone 3 on lysine 4 (H3K4 me 1/2/3). Through this activity, KMT2A acts as a transcriptional regulator of gene activity: H3K4 methylation is associated with an open chromatin conformation and active gene transcription. KMT2A exerts several important functions in the control of normal hematopoiesis, particularly at the level of the HSC compartment. Thus, *KMT2A* is required for HSC maintenance and self-renewal, as shown by experiments of *KMT2A* gene deletion showing a severe impairment of HSC proliferation and long-term repopulating capacity [5]. At the molecular level, *KMT2A* acts as a transcriptional coactivator, maintaining the expression of a set of genes, such as HOXA9, HOXA7, *MEIS1,* and PRDM16, which are crucial for the development and homeostatic regulation of hematopoietic stem and progenitor cells [5]. *KMT2A* also exerts an important role in the regulation of hematopoietic differentiation through control of the Rac/Rho/integrin signaling pathway. Finally, *KMT2A* exerts an important role in the cell cycle control and in the control of genomic stability, a function fundamental for the HSC compartment [10].

**Figure 2:**
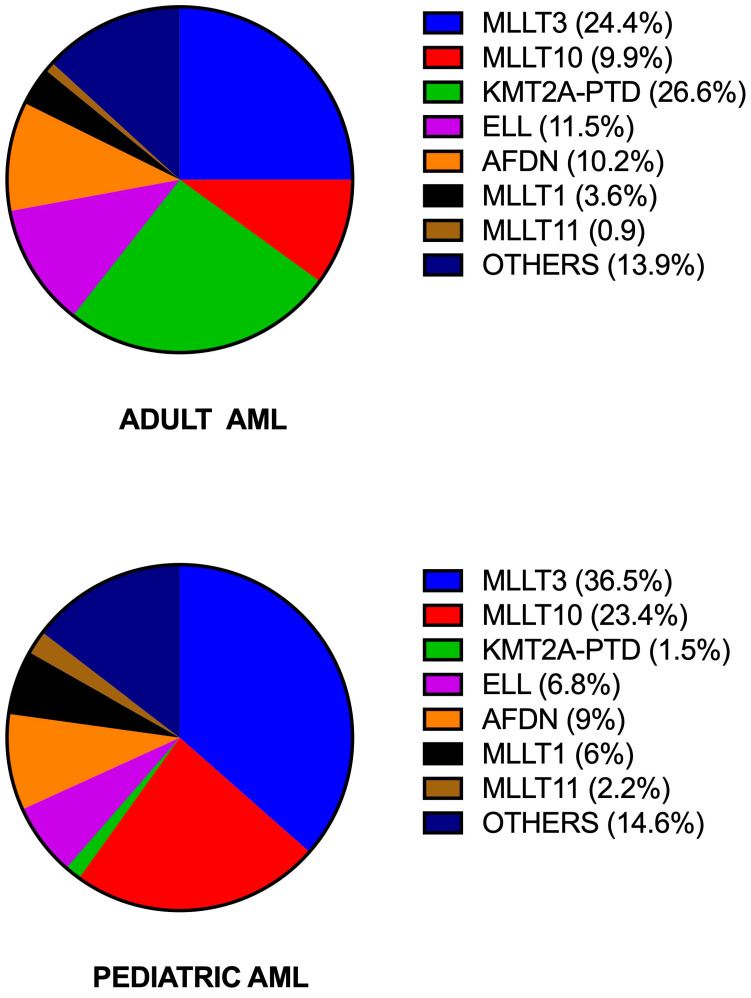
Frequency of the most recurrent *KMT2A* rearrangements observed in adult and pediatric AML patients with *KMT2A-r*. Data are reported in [11].

In acute leukemias, chromosomal rearrangements generate KMT2A fusion genes and the corresponding proteins in which the N-terminus of the *KMT2A* gene is fused in-frame to one of many different partners. There are over 90 unique oncogenic fusion partners that have been documented [8,9] (Figure 1). Frequent *KMT2A* rearrangements are represented by t(9;11)/*KMT2A::MLLT3*, t(6;11)/*KMT2A::AFDN*, t(11;19)(q23;p13.1)/*KMT2A::ELL*, t/ins(10;11)(p13;q23)/*KMT2A::MLLT10*, t(11;19)(q23;p13.3)/*KMT2A::MLLT1,* and t(11;17)(q23;q25)/*KMT2A::SEPTIN*; *KMT2A-PTD*, most commonly involving exons 2 to 9, with duplication of CxxC and ATH domains, transforming despite the absence of a fusion partner [11] (Figure 2).

The formation of KMT2A fusion proteins determines a condition of loss of function and gain of function: in fact, the fusion KMT2A protein loses its C-terminal SET domain and then loses its original H3K4 methyltransferase activity, but it gains a new potent C-terminal partner. Thus, the chimeric protein uses the retained N-terminal Menin-binding domain to bind to target promoters and uses the partner component, such as the Super Elongation Complex (SEC), to activate high-level, deregulated transcription of *HOX-A* genes.

Several studies have investigated the mechanisms through which *KMT2A* fusion genes promote leukemia development. According to these studies, *KMT2A* fusions can be categorized in five functional groups: direct AEP recruiter type (the fusion protein recruits an AEP complex on AF4 protein family), an EN family protein and p-TEFb (positive Transcription Elongation Factor b, a protein complex formed by CDK9 and cyclin T1 or T2); acetyl marker provider type; ENL provider type; multimerization type; and partial tandem duplication type [12]. In most of these different categories, there is evidence that the *KMT2A* fusion proteins act as conditionally active transcriptional regulators, involving *HOX-A9* upregulation and constitutive recruitment of AEP [12].

*KMT2A-PTD* is an intragenic, in-frame mutation where N-terminal exons, usually ranging from 2 to 10, are duplicated and inserted in tandem, resulting in a protein with a duplicated N-terminal DNA-binding domain. Breakpoints frequently occur in intronic regions flanking exons 2 to 10, causing a direct repeat, and the duplication typically encompasses the Menin-binding domain and the CxxC domain. The PTD is an internal tandem duplication. The N-terminal domains are translated in tandem, followed by the rest of the protein, not tacked on the end. The KMT2A-PTD protein retains the C-terminal portion of the full-length protein, including the SET domain. This differentiates KMT2A-PTD from KMT2A fusion proteins, which typically lose the SET domain.

*KMT2A* gene rearrangements can be detected by chromosome binding analysis and confirmed by fluorescence in situ hybridization (FISH). However, *KMTA-PTD* is too small to be detected by karyotype or FISH. The detection of *KMT2A-PTD* requires different technologies, such as next-generation sequencing (NGS), multiplex ligation probe amplification (MLPM), and optimal genome mapping (OGM) [13].

The mechanism through which *KMT2A-PTD* promotes AML development seems to be different from that mediated by KMT2A fusion proteins: in fact, KMT2A-PTD oncoprotein drives AML expression through a molecular mechanism involving ENL but not Menin [14]. This finding had important implications at therapeutic levels, in that *KMT2A-PTD* is characterized by a relative intrinsic insensitivity to Menin inhibitor monotherapy, which is mediated by a duplication of the CxxC domain and AT hooks of *KMT2A*. A concomitant inhibition of ENL and AF9YEATS domain, together with a Menin inhibitor, seems to be a strategy in *KMT2A-PTD* AML [14].

The key pathogenetic event operating in AML with *KMT2A* alterations is represented by uncontrolled, deregulated *HOX-A* gene expression. In normal hematopoiesis, the expression of *HOX-A* genes is finely tuned by a regulatory network implying activation by the KMT2A complex and repression by the PRC2 complex [15]. AML disrupts this balance, resulting in the persistent activation of *HOX-A*-regulated genes. The deregulated *HOX-A* expression promotes a leukemic condition by activating a set of target genes that stimulate proliferation, inhibit differentiation, and promote survival through inhibition of apoptosis [15]. This dysregulation may be related to different genetic events represented by *KMT2A-r*, *NPM1* mutations, and *NUP98-r,* all leading to persistent activity of *HOX-A* [15].

KMT2A fusion oncoprotein lacks the catalytic SET domain, and the KMT2A SET domain from the wild-type allele is dispensable in *KMT2A-r*; higher H3K4me3 levels are observed in leukemic stem cells and are required for the maintenance of these cells in an undifferentiated condition [16]. A recent study showed that SETD1B (KMT2G) is required for mediating H3K4me3 methylating activity in *KMT2A-r* AML cells and for the oncogenic activity of KMT2A fusion proteins [17]. Inactivation of SETD1B in *KMT2A-r* cells inhibits the amplitude of histone methylation and *MYC* gene expression [12]. SETD1B may represent a therapeutic target in *KMT2A-r AML* [17].

## 3. AML with KMT2A Rearrangements

*KMT2A-r* in AML displays an inverse relationship with age, appearing most frequently in infants (being observed up to 50–60% of cases in children <2 years) and decreasing to 5–15% in older children and 2.7% in adults [18].

The frequency of the different *KMT2A* translocations varied with age of AML patients: *KMT2A::MLLT10* and *KMT2A::MLLT11* fusions are more frequent in pediatric than in adult patients; *KMT2A::AFDN* is less frequent in pediatric than in adult patients; *KMT2A::MLLT3* and *KMT2A::ELL* fusions are similarly frequent in pediatric and adult patients; *KMT2A-PTD* is markedly less frequent in pediatric than in adult patients [11] (Figure 2).

Several recent studies have provided a characterization of pediatric *KMT2A-r* AML. Bolouri et al. (Children Oncology Group) reported the characterization of almost 1000 pediatric AML patients; in infants (<3 years), *KMT2A-r* was the most frequent abnormality [18]. The analysis of the mutational profile showed that *KMT2A-r* AML is characterized by a lower number of mutations compared to the rest of AML without this abnormality; *RAS*-pathway mutations are frequently associated with *KMT2A-r* AML [18].

Yuen et al. reported the characterization of 493 pediatric AML patients, including 105 *KMT2A-r* AML [19]. *KMT2A-r* AML were characterized by a younger age (median age 3.1 years) and by the presence of a higher rate of *NRAS*, *KRAS*, *PTPN11*, and *SETD2* mutations and a lower rate of *KIT*, *WT1*, and *FLT3-ITD* mutations [19]. *KRAS* and *SETD2* mutations were associated with *KMT2A-MLLT10* translocation [19]. *SETD2* mutations cooperate with *KMT2A-r* to promote leukemia development and confer chemoresistance through altered cell cycle control [20].

The prognostic implications of different *KMT2A* translocations observed in pediatric AML patients are variable: *KMT2A* translocations associated with *KMT2A::AFF1*, *KMT2A::AFDN*, *KMT2A::MTTLT10*, *KMT2A::ABI1,* and *KMT2A::MLLT1* exhibited a poor prognosis than the rest of *KMT2A* translocations; *KMT2A::MLLT11* had an intermediate risk, and *KMT2A::MTT3* had a favorable risk [21]. The high-risk group of childhood *KMT2A-r* AML had inferior EFS and OS and a higher cumulative incidence of relapse (CIR) than the non-high-risk group [16]. Allo-HSCT in high-risk *KMT2A-r* pediatric AML with flow cytometry MRD negativity at the end of induction 2, but not of induction 1, was associated with improved OS and EFS compared to MRD positivity [22].

A recent study on a large set of Japanese pediatric AML patients explored the mutational profiles in 59 *KMT2A-r* AML infants (<1 year) and 180 *KMT2A-R* children (>1 year to 10 years); in infants, *KMT2A-r* AML represent 24.7% of all pediatric *KMT2A-r* AML, and non-*KMT2A-2* AML only 2.4% of all pediatric non-*KMT2A-r* AML [23]. EFS and OS were significantly better in infant than in child *KMT2A-r* patients, while the opposite was observed for non-*KMT2A-r* pediatric AML [18]. *KMT2A::MLLT3* fusions were more frequent in children than in infant patients, with *KMT2A-ELL* fusions and other fusions being more frequent in infant than in child patients [18]. Signaling pathway mutations (mainly represented by *RAS* pathway mutations) are similarly frequent in infant and child patients; in contrast, non-signaling pathway mutations and, particularly, mutations of genes involved in epigenetic regulation are more frequent in child than in infant patients [18]. The presence of *KMT2A::MLLT4* fusions was associated with particularly poor prognosis among child patients; both infant and child patients with *KRAS* mutations have reduced EFS and OS; non-signaling mutations had no significant impact on the prognosis in either infants or children [23]. The study of 225 pediatric AML patients with *KMT2A-r* identified *KRAS* mutations as poor prognostic factors; particularly, *KRAS* codon G12 mutations were associated with a poorer prognosis when compared with other *KRAS* mutations [24].

Hernandez-Sanchez reported the genomic characterization of 205 adult *KMT2A-r* AML patients. In these patients, the most frequent translocations were t(9;11) (49%), t(11;19) (16%), t(6;11) (12%), t(10;11) (5%), and t(11;17) (5%) [25]. Additional cytogenetic abnormalities were present in 40% of these patients: complex karyotype (19%), trisomy 8 (18%), and trisomy 21 (5%) [25]. The most frequent gene mutations co-occurring with a *KMT2A-r* were: *NRAS* (21%), *KRAS* (19.5%), *FLT3-TKD* (13.3%), *TP53* (8.6%), *TET2* (8.1%), *ASXL1* (7%), *WT1* (7%), *DNMT3A* (6.5%), and *FLT3-ITD* (5.8%) [20]. RAS pathway signaling mutations *(NRAS*, *KRAS*, *PTPN11*, *BRAF)* were present in 42.1% of patients [25]. The mutational spectrum was similar for different *KMT2A* rearrangements [25].

Batayneh and workers reported the analysis of the mutational profile of 521 adult AML patients with *KMT2A-r* compared to 3863 *KMT2A*-WT patients [6]. *KMT2A-r* cases displayed a significantly increased frequency of *FLT3*, *KRAS,* and *IDH2* mutations compared to *KMT2A*-WT cases [19]. *KMT2A*-WT AML had a significantly increased frequency of mutations in *RUNX1*, *ASXL1,* and *TET2* [6].

Wu et al. reported the molecular characterization and outcomes of 180 adult *KMT2A-r* AML patients. *KRAS*-mutated patients had significantly worse two-year OS and higher two-year cumulative incidence of relapse (CIR) than WT patients (24.6% vs. 50.9% and 56.3% vs. 34.3%, respectively). *KRAS*-mutated patients had significantly lower two-year OS and higher two-year CIR than WT patients after transplantations (32.3% vs. 72.9% and 73.6% vs. 23.1%, respectively) [26].

## 4. Standard Treatment of AML Patients with KMT2A Rearrangements

### 4.1. Response in Adult AML with Different KMT2A Rearrangements and Co-Mutations

Bill and coworkers reported the molecular characterization and the outcomes of 172 adult AML patients with *KMT2A-r* [27]: 44% of patients had *KMT2A::MLLT3* fusions, 17% *KMT2A::AFDN6*, 12% *KMT2A::ELL*, 6% *KMT2A::MLLT1*, 8% *KMT2A::MLLT10*, 3% *KMT2A::SEPIN9,* and 10% other *KMT2A-r* [27]. Patients with *KMT2A-r* displayed a low number of additional gene mutations, mainly involving the *RAS* pathway *(NRAS, KRAS,* and *PTPN11*) [27]. RAS pathway mutations were significantly more frequent in patients with *KMT2A::AFDN* rearrangements [27]. Younger patients with *KMT2A::MLLT3* fusion genes had better outcomes than patients with other *KMT2A-r;* however, outcomes of older AML patients with *KMT2A/MLLT3* rearrangements were poor [27]. These observations suggested that the fusion partner of *KMT2A-r* influenced outcomes.

Hernadez-Sanchez et al. explored the outcomes of 205 adult AML patients with *KMT2A-r*, characterized by their mutational profile by NGS [25]. Overall survival of these patients was similar across the different *KMT2A* translocations, including those generating *KMT2A/MLLT3* fusion; however, t(9;11)(p21.3;q23.3)/*KMT2A::MLLT3* AML had an almost significant improvement of RFS compared to other *KMT2A* translocations [25]. Independent prognostic factors for OS were age > 60 years, secondary AML, and *KRAS* and *DNMT3A* mutations; in the subset of patients with de novo AML < 60 years, *KRAS* and *TP53* were the most prognostically relevant mutated genes, since patients with mutations of any of these genes had a lower CR rate (50% vs. 86%) and shorter mOS (7 vs. 30 months) [25]. Allo-HSCT in first CR improved OS [25].

### 4.2. Induction Chemotherapy with FLAG-IDA

Othman and coworkers reported the results of the analysis of 217 AML patients with *KMT2A-r* reported in the AML17 and AML19 studies, prospective randomized clinical trials of intensive chemotherapy for younger adults with ND AML, which incorporated several randomizations. In the whole treated population, CRc was 82%, mOS was 2.1 years, and three-year OS was 43%; relapse rate at three years was 47% in patients achieving a CRc [28]. In the AML19 study, AML patients were randomized to receive Tludarabine, Cytarabine, Idarubicin, and G-CSF (FLAG-IDA) or DA (Daunorubicin/Cytarabine): relapse rate (RR) was significantly lower with FLAG-IDA than with DA (three-year RR 26% or 68%, respectively), and there was a clear trend of improved OS for patients with FLAG-IDA compared to DA (three-year OS 66% vs. 37%, respectively) [28]. In the AML17 study, AML patients were randomized to receive DA or ADE (DA + Etoposide): the addition of Etoposide to DA did not improve relapse rate or survival over DA [28]. Patients who achieved a MRD-negative status after FLAG-IDA had particularly promising outcomes, with a three-year OS of 92% [28].

In line with these findings, Di Nardo and coworkers, in the context of a single-center study aiming to evaluate the safety and the effectiveness of a FLAG + IDA + Venetoclax regimen in ND and R/R AML patients, reported the results observed in nine AML patients with *KMT2A-r* AML: all patients achieved a CR, had undetectable MRD, with a mOS and mEFS not reached, and a three-year OS of 71%; and seven patients transitioned to allo-HSCT [29]. However, for R/R *KMT2A-r* AML patients, the responses were poor, with a CRc rate of 33% [29].

Zheng and coworkers have retrospectively evaluated the outcomes of 875 pediatric AML patients who received frontline therapy with FLAG + IDA (681 patients) or Daunorubicin, Ara-C, and Etoposide (DAE, 194 patients); in the whole population of patients, FLAG + IDA treatment significantly improved five-year OS over DAE treatment (79.6% vs. 69.3%, respectively) [30]. The benefit deriving from FLAG + IDA treatment compared to DAE treatment was evident in the group of patients with *KMT2A-r* AML (80.8% vs. 61.3%, respectively) [30].

### 4.3. Induction Therapy with Intensive Chemotherapy Plus Venetoclax

McMahon et al. reported a real-world retrospective study in 325 AML patients with *KMT2A-r* AML treated at both academic and community sites: 71% received intensive chemotherapy (IC), 17% Venetoclax (VEN) + a hypomethylating agent (HMA), and 11% HMA monotherapy; 42% underwent allo-HSCT [31]. Patients receiving treatment with IC had superior outcomes compared to patients receiving VEN + HMA: CRc rate (75% vs. 37%, respectively), three-year DFS (34% vs. 18%, respectively), and three-year OS (36% vs. 19%, respectively) [31]. In a retrospective analysis on 34 *KMT2A-r* AML patients, a significantly lower response rate was observed in patients treated with VEN + Azacitidine compared to those treated with IC [32].

Khaire et al. reported the results of a retrospective study carried out on 22 *KMT2A-r* newly diagnosed AML patients treated either with CLIA (Cladribine, Idarubicin, and Cytarabine) or CLIA + VEN: 35% of patients received CLIA (median age 51 years) and 65% of patients received CLIA + VEN (median age 41 years); 16% of the patients of the CLIA arm had t-AML and 25% of patients of the CLIA + VEN arm had t-AML [33]. In the CLIA arm, the CRc rate was 83%, while in the CLIA + VEN arm, it was 100%. In the CLIA arm, 60% of responders underwent allo-HSCT, and in the CLIA + VEN arm, it was 96%. The two-year EFS was 81% and 50% for CLIA and CLIA + VEN, respectively [33].

**Figure 3:**
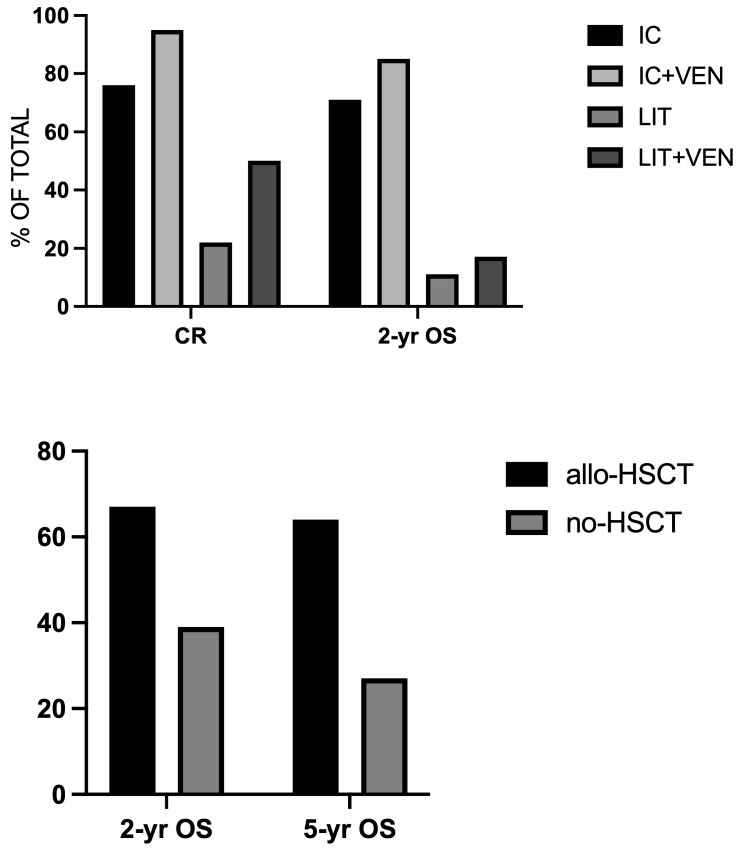
Top Panel: CR rate and two-year OS in *KMT2A-r* AML patients treated with intensive chemotherapy (IC), either alone or in combination with Ven (IC + VEN), or with low-intensity treatments (LIT), either alone or in combination with VEN. Bottom Panel: two- and five-year OS of *KMT2A-r* patients who have or have not undergone allo-HSCT. Data are reported in [34].

A recent retrospective analysis by Bataller et al. in a group of 1611 patients with ND AML showed *KMT2A-r* in 4.3% of cases. Patients treated with IC achieved a CRc rate of 81% and, when combined with VEN, the CRc rate was 100% [34] (Figure 3). Patients with low-intensity treatment (LIT) achieved a CRc rate of 33% and, when combined with VEN, the CRc rate increased to 61% (Figure 3). For patients treated with IC, the two- and five-year OS and EFS were 66% and 64%, respectively, compared with 7% in those treated with LIT [34]. Further, 37% of patients underwent allo-HSCT; patients who underwent an allo-HSCT in CR1 had an improved survival compared to those who did not undergo HSCT (two-year OS 67% vs. 39%, respectively); after five years, the OS of patients who received HSCT was 64% [34] (Figure 3). For patients treated with LIT, the presence of *NRAS* or *KRAS* was a negative prognostic factor [30]. For patients treated with IC only, the bone marrow blast percentage was the only variable predicting for OS and EFS [34]. For patients treated with IC, there was no significant difference between patients with *KMT2A-MLLT3* rearrangement and other rearrangements [34].

Older *KMT2A-r* AML patients treated with conventional therapy have a poor prognosis, with a mOS of <5 months [35].

### 4.4. Allo-Hematopoietic Stem Cell Transplantation in KMT2A-r AML Patients

Allo-HSCT is of fundamental importance to ensure the potential long-term survival of *KMT2A-r* AML patients. Chen and coworkers reported the study of 125 AML patients median age 51 years) with *KMT2A* alterations, including 45 with *KMT2A-r*, 64 with *KMT2A-PTD,* and 14 with both *KMT2A-r* and *KMT2A-PTD* [36]. As expected, the mutational profile of *KMT2A-r* and *KMT2A-PTD* AML was different; patients with both *KMT2A-r* and *KMT2A-PTD* had genetic profiles more closely resembling those of the *KMT2A-r* group [36]. The majority (77%) of patients were treated with IC, and a minority (23%) with reduced-intensity therapy, and their OS and EFS were similar [36]. Patients were stratified into three risk groups: intermediate risk (*KMT2A-MLLT3* and *KMT2A-ELL*), high risk (*KMT2A-PTD*), and very high risk (*KMT2A-AFDN* and other *KMT2A-rs*), with different three-year OS (78%, 51%, and 35%, respectively) [36]. Sixty-eight patients proceeded to allo-HSCT, markedly improving their survival: three-year OS 78% with HSCT and 23% without HSCT, and three-year EFS 66% with HSCT and 12% without HSCT [36].

Alzarkali and coworkers explored a group of 81 *KMT2A-r* AML patients treated at the Moffitt Cancer Center (Tampa, USA) who underwent either first-line IC (69 patients) or low-intensity therapy (12 patients) [37]. The CRc rate was 86.6% for IC and 36.4% for LIT [37]. Thirty-four patients received allo-HSCT, and their median OS and PFS were 93.8 and 82.1 months, respectively, compared to 11.5 months and 5.3 months, respectively, for those who did not receive HSCT [37].

Shen et al. have evaluated the outcomes of 181 *KMT2A-r* AML who received IC treatment; 74% of patients underwent allo-HSCT in CR1 [34]. The patients who received allo-HSCT had a clearly better OS and EFS compared to those not receiving transplantation [38]. The benefit deriving from allo-HSCT was markedly more evident for patients with age > 20 years compared to those with age < 20 years [38]. All the most recurrent *KMT2A-r* had a benefit from allo-HSCT, but the OS post-transplantation was different for different *KMT2A-r* [38].

In *KMT2A-r* AML patients, the MRD status post-induction chemotherapy is a major predictor of outcomes of allo-HSCT. Thus, Loo et al. showed, in a cohort of 54 *KMT2A-r* patients who underwent allo-HSCT after achieving a CRc post-induction therapy, that the presence of a measurable MRD pre-transplant was associated with inferior post-transplant outcome [39]. Wang et al. confirmed that residual *KMT2A-r* before allo-HSCT predicts the risk of survival and relapse and suggested that donor lymphocyte infusion or post-transplantation maintenance therapies must be considered for patients with detectable MRD [40]. Zhang et al. reported the analysis of 292 *KMT2A-r* AML patients, of whom 87% achieved a CR, and 75.6% of responding patients underwent allo-HSCT [40]. These patients did benefit from allo-HSCT in first CR, and in transplanted patients, MRD evaluation predicted transplantation outcomes [41]. Molecular-based quantification of *KMT2A-r* MRD prior to allo-HSCT is a better surrogate for transplant prognosis than multiparameter flow cytometry-based MRD assessment [41].

Liu et al. monitored *KMT2A-r* gene expression at various times after chemotherapy: post-induction (MRD1), post-first consolidation (MRD2), and post-second consolidation (MRD3); the incidence of MRD negativity peaked at MRD2 [42]. The study of 52 *KMT2A-r* patients who underwent allo-HSC showed that *KMT2A-r* status after chemotherapy and its kinetics are significant HSCT prognostic indicators [42].

The studies of allo-HSCT in *KMT2A-r* AML patients have shown that HSCT determines a substantial improvement in the long-term survival and that the presence of a remission status prior to HSCT is a crucial factor influencing the outcomes. Patients who did not achieve remission before HSCT faced significantly poorer outcomes, highlighting the need for effective pre-transplant therapies to induce remission.

As discussed above, *KMT2A-PTD* displays immunophenotypic and molecular features different from *KMT2A-r* AML. A recent study showed that morphological and immunophenotypical features typically described in myelodysplasia are observed in about 40% of *KMT2A-PTD* AML; *IDH2*, *FLT3*, *RUNX1,* and *DNMT3A* mutations were frequent in these leukemias [43]. Furthermore, 44% of AML with *KMT2A-PTD* could be diagnosed as AML-MR based on cytogenetics or genomics [43]. The outcomes of *KMT2A-PTD* with or without MR-associated abnormalities were similar.

*KMT2A-PTD* AML showed a reduced RFS and OS compared to *KMT2A-WT* AML [44]. In fact, Kunadt retrospectively analyzed the outcomes of 136 *KMT2A-PTD* patients and 856 WT AML patients receiving intensive induction therapy [44]. Five-year RFS and OS were lower for *KMT2A-PTD* than for WT patients (15% vs. 25% and 28% vs. 51%, respectively) [44]. *KMT2A-PTD* patients who proceeded to allo-HSCT, compared to *KMT2A-PTD* patients who received only consolidation chemotherapy, showed an improvement of five-year OS and mOS from 33% to 47% and from 30 months to 43 months, respectively [44]. Allo-HSCT significantly improved both RFS and OS in these patients, as confirmed by several studies [44,45].

## 5. Target Therapy of *KMT2A-r* AML

Molecular studies on the mechanisms of action of KMT2A fusion proteins and preclinical studies have strongly supported the clinical use of Menin inhibitors.

**Figure 4:**
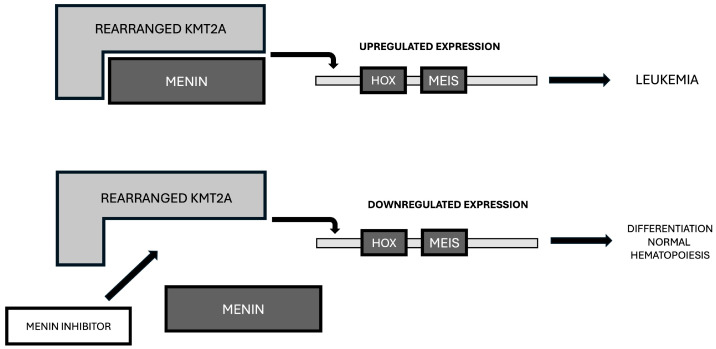
Mechanism of action of Menin inhibitors. Top panel: In *KMT2A-r* leukemic cells, the molecular complex of KMT2A fusion protein with Menin binds to chromatin and stimulates the overexpression of *HOX-A9* and *MEIS* genes, driving leukemic development. Bottom panel: The addition of a Menin inhibitor binds to Menin, impeding its interaction with KMT2A fusion protein and, consequently, downregulates *HOX-A9* and MEIS expression and induces differentiation and apoptosis of leukemic cells.

High-throughput screening studies have led to the identification of several compounds that act as small-molecule inhibitors of the interaction between *KMT2A-r* and Menin or *NPM1m* and Menin. Menin inhibitors disrupt this protein-protein binding and, consequently, block the expression of genes such as *HOXA9* and *MEIS1*, forcing the differentiation and apoptosis of leukemic cells [46]. Physiologically, Menin acts as a scaffold protein that supports the binding of KMT2A fusion proteins or NPM1m protein to DNA; Menin inhibitors occupy the pocket binding of Menin, thus blocking their capacity to interact with *KMT2A-r* or *NPM1m* [42]. By disrupting the binding of the complex KMT2A-rearranged protein-Menin to chromatin, Menin inhibitors induce a rapid downregulation of *HOXA9* and *MEIS1* gene expression, with consequent induction of leukemic cell differentiation and apoptosis (Figure 4).

Four Menin inhibitors, Ravumenib, Bleximenib, Enzomenib, and Ziftomenib have been selected for their potent inhibitory activity and have been evaluated in AML patients with *KMT2A-r*, *MPM1m*, and *NUP-98-r*. Two of these compounds have been approved for clinical use.

### 5.1. Monotherapy Studies with Menin Inhibitors

Several clinical trials have explored the safety and the effectiveness of monotherapy studies with Menin inhibitors in *KMT2A-r* AML patients (Table 1).

#### 5.1.1. Monotherapy Studies with Revumenib

In a phase I clinical study, treatment with Revumenib showed an acceptable profile of safety and provided preliminary evidence about the effectiveness of Revumenib in patients with *KMt2A-r.* The pivotal phase II, registration-enabling portion of the clinical study AUGMENT-101 involved 94 patients with *KMT2A-r* acute leukemia, including 78 patients with AML, 14 with ALL, and 2 with acute leukemia of ambiguous lineage, with a mean age of 37 years [47]. At the level of safety, grade ≥ 3 adverse events included febrile neutropenia (37%), differentiation syndrome (16%), and QTc prolongation (13.8%) [47]. The CRc rate was 22.8%; 70% of patients who achieved a CRc condition were MRD-negative by flow cytometry; the ORR was 63%; the median duration of CR was 6.4 months; and the median OS was 8 months [47]. Analysis of the transcription profile of bone marrow cells of treated patients showed a significant downregulation of target genes *MEIS1*, *HOXA9*, and *PBX3,* and an upregulation of the genes associated with cell differentiation, such as *CD11b* and *CD14* [47]. The analysis of the outcomes of the 78 *KMT2A-r* AML patients enrolled in the AUGMENT-101 study showed an ORR of 67%, a CRc rate of 23%, a median DOR of 7.7 months, and a MRD-negativity of 64% among patients achieving a CR; 19 patients proceeded to allo-HSCT [48]. According to the results of this study, in November 2024, the FDA approved the clinical use of Revumenib as monotherapy in R/R *KMT2A-r* AML patients.

A recent study reported the immunophenotype of leukemia cells by flow cytometry in 48 *KMT2A-r* AML patients treated with Revumenib; dynamic changes in the immunophenotype after treatment were observed in 52% of patients, characterized by a switch from a myeloid/stem-like to a monocytic or myelo-monocytic immunophenotype, or by substantial changes in the intensity of antigen expression or patterns of leukemia-associated immunophenotypes [45]. Morphologic remission with MRD-negativity by flow cytometry following Revumenib was associated with improved OS [49].

In spite of the consistent effectiveness of Revumenib in the treatment of R/R *KMT2A-r* AML patients, 40% of patients treated with Revumenib monotherapy developed Menin inhibitor resistance through different molecular mechanisms dependent on or independent of *Menin1* (*MEN1*) mutations. Thus, it was shown that leukemic cells with *KMT2A-r* may acquire somatic changes in *MEN1* structure, reducing the effectiveness of Menin inhibitors; in fact, mutations at the level of the residues H327, G331, T349, and S160 reduce the ability of Revumenib to interact with Menin, without affecting the capacity of Menin to interact with KMT2A [50]. Consequently, the Menin-KMT2A fusion proteins continue to activate their target genes and to drive leukemogenic expression, despite exposure to the Menin inhibitor [50]. In KMT2A patient-derived xenograft model (PDX), the continuous exposure to Menin inhibitors generated *MEN1* mutations in 80% of mice treated with lower dose Menin inhibitor therapy; with higher doses of Menin inhibitor, only 20% of mice developed a *MEN1* mutation and in the remaining mice *MEN1*-WT cells persisted and slowly expanded over six months of therapy, despite the on-target gene expression changes [51]. These observations suggest remarkable differences in resistance mechanisms dependent on Menin inhibitor dose.

Studies on *KMT2A-r* AML patients treated with Revumenib, as well as in murine models of *KMT2A-r* leukemia, showed that *TP53* inactivation is associated with resistance to this Menin inhibitor, through a mechanism seemingly related to upregulation of the antiapoptotic MCL1 protein [52].

Soto-Feliciano and coworkers showed the existence of a resistance mechanism to Revumenib not dependent upon *MEN1* mutations [52]. In fact, these authors showed that Menin-KMT2A interaction promotes leukemia survival, inhibiting the binding of the KMT2B/C-UTX complex to target gene promoters; Revumenib, disrupting the MEN-KMT2A interaction, triggers UTX-dependent transcriptional activation of a tumor suppressive program required to confer therapeutic response in *KMT2A-r* leukemic cells [40]. In Revumenib-resistant *KMT2A-r* AML patients, there is a loss of activation of this pathway, and this activity can be rescued using CDK4/6 inhibitors [53].

These cases of non-genetic Menin inhibitor resistance showed a marked reprogramming of gene expression and, at variance with resistant leukemic cells with *MEN1* mutations, displayed a maintained capacity of the Menin inhibitor to displace Menin from chromatin [54]. Using a genome-wide CRISPR-Cas9 screen, it was shown that inactivation of the histone acetyl transferase KATA6 was able to reverse the resistance phenotype and restore sensitivity to the Menin inhibitor [54]. A recent study provided clear evidence that KATA6 and KATA7 interact with Menin and the KMT2A complex and are colocalized at the level of chromatin regions where they coregulate oncogenic transcriptional programs [55]. The functional significance of these observations is supported by experiments of double KAT6A and KAT7 inhibition using the PF-9363 inhibitor, eliciting eviction of the KMT2A fusion protein from chromatin, potent repression of oncogenic transcription, and overcoming of primary resistance to Menin inhibitors [55].

#### 5.1.2. Monotherapy Studies with Enzomenib

Enzomenib (ENZO) is an oral small-molecule inhibitor of the Menin and KMT2A interaction with a short half-life of 2–5 h, low lipophilicity, and high clearance. A phase I-II dose-escalation/optimization study evaluated ENZO in monotherapy in 116 R/R AML patients (108 AML, 61 *KMT2A-r,* and 34 with *NPM1m* AML), of which 31% had prior HSCT and 74% had prior VEN [56]. Treatment was well tolerated, with grade ≥ 3 in 7.7% of patients; grade 1–2 QTc prolongation was observed in 4.3% of cases [56]. For *KMT2A-r* patients treated with ENZO + azoles, the ORR rates and CRc rates at doses of 200, 300, and 400 mg were 50%, 16.7%, and 72.7%, and 45.5, 75, and 25%, respectively [56]. The duration of CRc at 300 mg was not reached; the median OS for all patients with *KMT2A-r* AML treated at ≥200 mg ENZO was 11.4 months [56].

#### 5.1.3. Monotherapy Studies with Ziftomenib

Ziftomenib, an oral selective Menin inhibitor, was evaluated in the multicenter phase I-II KOMET-001 clinical trial in adult patients with R/R *KMT2A-r* or *NPM1m* AML [44]. Phase I of this study was designed to evaluate the safety profile of Ziftomenib and to determine the optimal dose for phase II studies [57]. In phase I, 83 patients were enrolled, and no clinical responses were observed at the dose of 200 mg of Ziftomenib; at the 600 mg dose of Ziftomenib, 25% of *KMT2A-r* or *NPM1m* AML (12.5% in *KMT2A-r* and 35% in *NPM1m* AML) patients had CRc [57]. For the rate and severity of differentiation syndrome, the enrollment of patients with *KMT2A-r* was halted, and only *NPM1m* patients were evaluated in phase II of the study [57].

#### 5.1.4. Monotherapy Studies with Bleximenib

The phase I-II cAMELot-1 study evaluated the safety and the optimal dose for phase II of the oral Menin inhibitor Bleximenib; 121 patients with R/R acute leukemia (108 AML) were enrolled [54,55]. The optimal dose for phase II studies was estimated to correspond to 100 mg of Bleximenib. The most serious adverse event observed in these patients was differentiation syndrome (DS), with a frequency of 13% and with two cases of fatal differentiation syndrome; cardiac toxicity was very limited [58,59]. At the optimal dose tested (90 mg), the rate of CRc of 33.3% was observed both for *KMT2A-r* and *NPM1m* AML patients [59].

### 5.2. Menin Inhibitors in Combination with Chemotherapy

#### 5.2.1. Revumenib in Combination with Chemotherapy

Several clinical trials are evaluating Revumenib in association with intensive chemotherapy (IC), both in R/R and ND patients. The AUGMENT-102 clinical trial is a phase I dose-escalation study evaluating the safety, tolerability, and effectiveness of Revumenib in combination with chemotherapy (Fludarabine and Cytarabine) in children and adults with R/R *KMT2A-r* and *NPM1-m* AML [60]. The evaluation of the first 27 patients (mostly pediatric) showed a CRc rate of 50–55% [60]; 71.4% of patients who achieved CRc were MRD-negative, and many of them proceeded to allo-HSCT [60]. The adverse event profile was compatible with the background chemotherapy, and no cases of differentiation syndrome were reported [60].

A phase I clinical study is evaluating the safety and the effectiveness of Revumenib in combination IC (7 + 3 standard regimen) in patients with ND *KMT2A-r* and *NPM1-m* and *NUP98-r* [61]. Preliminary data in the first seven patients treated at the low Revumenib dose (DL1) showed a safety profile compatible with known safety profiles of IC and Revumenib; the CRc rate was 100%, with 100% MRD negativity [61].

#### 5.2.2. Ziftomenib in Association with Chemotherapy

The KOMET-007 study is an ongoing dose-escalation/expansion clinical study evaluating Ziftomenib with standard chemotherapy in *KMT2A-r* and *NPM1-m* AML. The patients were treated with Ziftomenib (600 mg) plus 7 + 3 IC (Cytarabine/daunorubicin) induction, then consolidation with Cytarabine and/or allo-HSCT [62]. In January 2025, 51 ND AML patients with *KMT2A-r* (16 patients) and *NPM1-m* (35 patients) AML were treated [62]. Adverse events were those expected for this type of treatment. CRc rates were 94% for *NPM1-m* and 83% for *KMT2A-r* patients; with a median follow-up of 19.7 weeks, OS rates were 97% for *NPM1-m* and 83% for *KMT2A-r* AML [62]. No cases of differentiation syndrome were observed.

#### 5.2.3. Bleximenib in Association with Chemotherapy

The ALE 1002 phase Ib study explored the safety and the effectiveness of the combination of Bleximenib with IC 7 + 3 in 44 ND AML patients with *KMT2A-r* (43%) or *NPM1-m* (57%) [63]. The safety profile was that expected for the type of treatment; no differentiation syndrome was observed; only three cases of QT prolongation of grade 1–2 were observed [63]. The ORR was 95.8% and the CRc rate 87.5%; responses were similar for both *KMT2A-r* and *NPM1-m* patients [63].

A randomized phase III HOVON 181 AML/AML SG 37-25 clinical trial is evaluating the combination of Bleximenib plus IC 7 + 3 vs. IC 7 + 3 in *KMT2A-r* and *NPM1-m* AML, with EFS selected as the primary endpoint [64].

### 5.3. Menin Inhibitors in Combination with Venetoclax

Several recent studies have explored the safety and the effectiveness of Menin inhibitors in combination with VEN (Table 2).

#### 5.3.1. Revumenib in Association with Venetoclax

Revumenib was evaluated in association with VEN both in R/R and ND *KMT2A-r* and *NPM1-m* AML patients. The SAVE study evaluated the safety and the effectiveness of the triplet combination of Revumenib, Decitabine/Cedazuridine, and VEN in R/R and ND *KMT2A-r* and *NPM1-m* patients. The study in R/R AML patients involved 26 patients (*KMT2A-r*, *NPM1-m*, and *NUP98-r*), sharing an ORR of 88%, with a CRc rate of 58%, and with 93% of MRD negativity among patients with CRc; with a median follow-up of six months, RFS was 59%, and OS was 74% [65].

The study carried out in 17 ND AML patients with either *KMT2A-r* (35%) or *NPM1-m* (65%) AML; the median age of patients was 68 years, and 24% had s-AML or t-AML [62]. CR rate was 88%, and 100% of patients who achieved CR were MRD-negative; all *KMT2A-r* patients were also negative by FISH analysis after treatment [66]. At six months of follow-up, the median EFS and OS were not reached, and 50% of *KMT2A-r* patients proceeded to allo-HSCT [66].

A recent phase I dose-escalation and expansion study evaluated Azacitidine (AZA), Ven, and Revumenib (at two dose levels, 113 mg or 163 mg) in 43 patients aged 60 years or older, with ND AML patients with *KMT2A-r* or *NPM1m* [63]. The safety profile was acceptable, with differentiation syndrome observed in 19% patients and QTc prolongation in 44% of patients, and neither required permanent discontinuation of Revumenib [67]. For *NPM1m* and *KMT2A-r* patients, ORR was 85.3% vs. 100%, CRc rate was 79.4% vs. 88.9%, CR rate was 65% vs. 78%, and MRD negativity by flow cytometry was 100% in 37 evaluable patients. Patients proceeding to allo-HSCT was 20.6% vs. 23.2%, respectively [67]. With a median follow-up of 6.9 months, median EFS, median OS, and one-year OS were 13.3 months, 15.5 months, and 62.9%, respectively; median OS was 15.5 months versus 18.0 months in *NPM1m* versus *KMT2A-r*, and, considering all patients, 17.0 months versus not reached at the lowest and at the highest Revumenib dose, respectively [67]. On the basis of these findings, a randomized phase III study is in development comparing Azacitidine, Venetoclax, and Revumenib with Azacitidine, Venetoclax, and a placebo in older/unfit patients newly diagnosed with *NPM1m* AML to determine whether the addition of Revumenib improves OS in this patient population.

#### 5.3.2. Zeftomenib in Association with Venetoclax

The study KOMET-007 explored the triplet combination of VEN, AZA, and Ziftomenib in both R/R and ND *KMT2A-r* and *NPM1m* AML patients. The study on R/R patients involved 80 patients (51 *NPM1m* and 29 *KMT2A-r*) [64]. Ziftomenib-related adverse events were limited, and 6% of patients discontinued treatment due to adverse events [68]. ORR was 65% for *NPM1m* and 33% for *KMT2A-r* patients; CRc rates were 49% for *NPM1m* and 22% for *KMT2A-r* AML, associated with 50% and 60% of MRD-negativity, respectively [68]. In VEN-naïve patients, CRc rates were 71% for *NPM1m* and 33% for *KMT2A-r* patients [68]. For ND AML patients, the results of the KOMET-0097 study are available only for *NPM1m* AML patients [69].

#### 5.3.3. Bleximenib in Association with Venetoclax

The ALE 1002 clinical study explored the safety and the effectiveness of Bleximenib in association with VEN in R/R *KMT2A-r* and *NPM1m* AML patients [66]. Fifteen patients received the combination of Bleximenib with VEN; the treatment was well tolerated, and one patient required dose adjustments [70]. ORR was 69%, with a CRc rate of 23%; responses were also maintained in patients who received prior VEN exposure; 30% of patients proceeded to allo-HSCT [70].

A phase Ib study evaluated VEN + AZA + Bleximenib at 15 to 150 mg (R/R) or 30 to 100 mg (ND) in 120 AML patients with *KMT2A-r* (52 patients) or *NPM1m* (68 patients) [71]. The safety profile was acceptable, with 4% of differentiation syndrome events and no QT prolongation events [67]. In the R/R group, ORR and CRc rates were lower, with 50 mg (76% and 32%) versus 100 mg (79% and 54%); in the ND group, ORR and CRc rates were lower with 50 mg (77% and 62%) versus 100 mg (92% and 85%) [71]. This triplet combination therapy showed an acceptable safety profile and promising efficiency, supporting additional exploration in the context of the phase III cAMELot-2 study.

#### 5.3.4. Enzomenib in Association with Venetoclax

A phase I clinical study evaluated Enzomenib in combination with VEN and AZA in 18 patients with R/R *KMT2A-r* and *NPM1m* AML; Enzomenib was evaluated at three different doses [72]. Enzomenib up to 300 mg was well tolerated in combination with VEN + AZA, with no dose-limiting toxicities; no QT prolongation was reported, and only one patient with grade 1–2 developed differentiation syndrome [68]. In the whole population of patients, ORR was 83% and CRc rate was 56%, with 86% of MRD negativity among responders [72]. In patients without VEN or MI exposure, ORR was 100%, with a CRc rate of 67% [72].

### 5.4. Menin Inhibitors in Combination with FLT3 Inhibitors

Preclinical studies have clearly shown the synergistic anti-leukemic effects of Menin inhibitors when added in combination with FLT3 inhibitors in *FLT3m KMT2A-r* or *NPM1m* AML. A significant proportion of *NPM1m* (ranging from 35% to 55%) and *KMT2A-r* (ranging from 10% to 30%) AML exhibit *FLT3* co-mutations; thus, there is a rationale in these patients to explore the therapeutic impact deriving from the combination of a Menin inhibitor with a FLT3 inhibitor. Thus, Borate and coworkers recently reported the preliminary results of a phase I study exploring the safety, tolerability, and effectiveness of the combination of Revumenib with Gilteritinib (a FLT3 inhibitor) [73]. The preliminary results observed in 7 R/R AML patients (five *NPM1m*, one *KMT2A-r*, and one *NUP98-r*) showed that Revumenib can be combined with Gilteritinib with encouraging preliminary efficacy [73].

### 5.5. Menin Inhibitors as Post-Transplant Maintenance Therapy in KMT2A-r AML

In the AUGMENT-101 trial, 39% of patients who achieved a response underwent allo-HSCT. Nine patients resumed Revumenib administration 59 to 180 days after HSCT; Revumenib dose was reduced for four of these nine patients to mitigate adverse events [65]. Revumenib duration of treatment in the maintenance setting ranged from 23 to 588 days, with treatment ongoing for five of these nine patients; CRc was maintained in six of these nine patients after HSCT and maintenance Revumenib [74]. Notably, one patient, who was MRD-positive after allo-HSCT, converted to a MRD-negative condition during maintenance therapy [74]. Cuglievan and coworkers reported the retrospective analysis of 10 *KMT2A-r* and two *NUP98-r* pediatric AML patients who have received first-line Revumenib monotherapy as maintenance therapy [75]. The patients received a median number of 11 cycles of maintenance therapy, with a 100% survival rate and no relapses, with a one-year EFS of 100% [75].

## 6. Conclusions

*KMT2A-r* AML are generally considered and classified as adverse- or intermediate- risk leukemias, characterized by reduced response to chemotherapy and a high likelihood of relapse. Progresses in induction treatments in patients fit for chemotherapy and in target treatments using Menin inhibitors have improved the outcomes of *KMT2A-r* AML patients.

A retrospective analysis by Bataller and coworkers showed the consistent improvements in OS observed in *KMT2A-r* AML patients from 1990 to 2022: two-year OS rates of 21% in the 1990–1999 decade, 19% in the 2000–2009 decade, 38.4% in the 2010–2019 decade, and 55% in the 2020–2022 [34]. The introduction of more effective induction treatments, such as FLAG-IDA and the association of VEN to IC regimens, was in part responsible for this improvement, particularly in recent years. The important contribution of this more efficacious induction IC regimens was related to an improvement in the rate and in the quality of remission achieved, offering an increased opportunity for more patients to proceed to allo-HSCT [30]. Furthermore, for patients treated with IC, there was no significant difference between patients with *KMT2A-MLLT3* rearrangement, historically considered more favorable, and those with other rearrangements considered more adverse [30]. The better effectiveness of this induction IC regimens, and their combinations with VEN or Menin inhibitors, if confirmed through randomized clinical trials, will modify the induction for first-line treatment of *KMT2A-r* AML patients [76].

Menin inhibitors have transitioned from an emerging targeted therapy to a keystone of treatment for AML patients with *KMT2A-r*, *NPM1m*, and *NUP-98-r*. Following the initial FDA approvals of Revumenib in late 2024 (R/R *KMT2A-r)* and 2025 (*R/R NPM1m*) and Ziftomenib in late 2025 (*R/R NPM1m*), the landscape of treatments based on Menin inhibitors is now shifting toward exploring these agents in combination with standard of care for adult and older patients, in earlier lines of therapy, as maintenance therapy post-HSCT and for overcoming resistance to Menin therapy.

While Menin inhibitors are effective in *KMT2A-r* AML, *KMT2A-PTD* AML often demonstrates resistance because the duplication (mostly of the CxxC/AT hooks) retains chromatin binding even after treatment. Due to potential resistance to single-agent Menin inhibitors, combining these inhibitors with other agents must be evaluated in future studies at experimental and clinical levels.

## Figures and Tables

**Table 1:** Major clinical trials involving the use of Menin inhibitors in monotherapy in R/R *KMT2A-r* and *NPM1m* AML patients.

Trial Name NCT Identifier Phase	Patient Number and Disease Status	Therapeutic Regimen	Efficacy	Toxicity
AUGMENT-101 NCT04065399 Phase I/II	Adult and pediatric patients with R/R NPM1m (116) and *KMT2A-r* (84) AML	Revumenib (single-arm)	*KMT2A-r* (116 pts) ORR 63.2% CRc 22.8% DOR 4.3 mo OS 8.0 mo NPM1m (84 pts) ORR 46.9% CRc 23.4% DOR 4.4 mo OS 4.0 mo	*KMT2A-r* DS 27% QTc prol 29% NPM1m DS 19% QTc prol 43%
KOMET-001 NCT04067336 Phase I/II	Phase Ib: patients with R/R NPM1m or *KMT2A-r* AML Phase II: patients with R/R NPM1m 200 mg 20 pts 600 mg 130 pts	Ziftomenib (single-arm)	*KMT2A-r* (18 pts) ORR 17% CRc 11% DOR 2.1 mo NPM1m (112 pts) ORR 33% CRc 25% DOR 3.7 mo OS 6.1 mo	All patients DS 14% QTc prol NR
DSP-5336-101 NCT04988555 Phase I/II	Phase I: patients with R/R Acute Leukemia Phase II: patients with R/R NPM1m or *KMT2A-r*	Enzomenib (single-arm)	*KMT2A-r* (300 mg 11 pts) ORR 72.7% CRc 45.5% DOR 4.3 NR OS 11.4 mo NPM1m (200–300 mg 17 pts) ORR 58.8% CRc 47% DOR 5.9–6.7 mo OS 8.5 mo	All patients (116) DS 12.9% QTc prol 4.3%
cAMELot-1 NCT04811560 Phase I/II	Phase Ib: patients with R/R NPM1m or *KMT2A-r* or NUP-98-r AML Phase II: patients with R/R NPM1m or R/R *KMT2A-r* AML	Bleximenib (single-arm)	*KMT2A-r* (90–100 mg 9 pts) CRc 33.3% NPM1m (90–100 mg 12 pts) CRc 33.3% All patients (146) DOR 6 mo	All patients DS 19.4% QTc prol NR

**Table 2:** Major clinical trials involving the use of Menin inhibitors in combination with Venetoclax in R/R and ND *KMT2A-r* and NPM1m AML patients.

Trial Name NCT Identifier Phase	Patient Number and Disease Status	Therapeutic Regimen	Efficacy	Toxicity
SAVE NCT005360160 Phase I	Adult patients with R/R NPM1m, *KMT2A-r*, NUP-98-r AML (26 patients)	Revumenib Venetoclax Decitabine/ Cedazuridine	ORR 88% CRc 43.9%	DS 4% QTc prol 8%
SAVE NCT005360160 Phase II	Adult patients with ND NPM1m, *KMT2A-r*, NUP-98-r AML (17 patients)	Revumenib Venetoclax Decitabine/ Cedazuridine	ORR 94% CR 88% 6-mo EFS 59% Median OS NR	DS 12% QTc prol 18%
BEAT AML Master Trial	Adult patients with ND NPM1m (34 patients) or *KMT2A-r* (9 patients)	Revumenib Venetoclax Azacitidine	*KMT2A-r* (9 pts) ORR 100% CRc 88.9% CR 78% OS 18 mo NPM1m (34 pts) ORR 85.3% CRc 79.4% CR 65% OS 15 mo	All patients DS 19% QTc prol 44%
KOMET-007 NCT05735184 Phase Ia/Ib	Patients with R/R NPM1m or *KMT2A-r* (80 patients)	Ziftomenib Azacitidine Venetoclax	*KMT2A-r* (29 pts) ORR 33% CRc 22% OS 21 wks NPM1m (51 pts) ORR 65% CRc 49% OS NR	All patients DS 12% QTc prol 0%
ALE1002 NCT05453903 Phase Ib	Patients with R/R NPM1m (10 patients) or *KMT2A-r* (3 patients)	Bleximenib Venetoclax	ORR 69.2% CRc 38.5%	All patients DS 0/13 QTc prol 1/13
ALE1002 NCT05453903 Phase Ib	Patients with NPM1m (68 patients) or *KMT2A-r* (52 patients) R/R (86 patients) or ND (34 patients)	Bleximenib Venetoclax Azacitidine	R/R (50/100 mg) ORR 76/79% CRc 32/54% ND (50/100 ng) ORR 72/92% CRc 62/85%	All patients DS 4% QTc prol 0%
Phase I	Patients with R/R NPM1m or *KMT2A-r* (18 patients)	Enzomenib Venetoclax Azacitidine	ORR 83% CRc 56%	All patients DS 0% QTc prol 1/18
